# Integrating services to improve the return-to-work process in depression or anxiety: results from a three-arm parallel randomized trial

**DOI:** 10.1192/j.eurpsy.2022.258

**Published:** 2022-09-01

**Authors:** A. Hoff

**Affiliations:** Capital Region Psychiatry, CORE - Copenhagen Research center for Mental Health, Capital Region Mental Health Services, KØBENHAVN N, Denmark

**Keywords:** integrated care, Depression, vocational rehabilitation, Anxiety

## Abstract

**Introduction:**

Depression and anxiety are very frequent and associated with high societal costs, much suffering and functional impairment. Employment is essential and pivotal recovery after sick-leave. In many countries, health care interventions are delivered separately from vocational rehabilitation services. This fragmented placement of interventions often implies lack of coordination, creating despair among sick-listed persons.

**Objectives:**

The aim of this trial was to investigate an integrated mental health care and vocational rehabilitation intervention to improve and hasten the return-to-work process among people sick-listed with anxiety or depression.

**Methods:**

In this RCT, participants were randomly allocated to A) integrated interventions (INT), B) improved mental health care (MHC) or B) service as usual (SAU). Primary outcome was time to return-to-work during 12-month. Secondary outcomes were time to return-to-work at 6-month follow-up; levels of anxiety, depression, stress symptoms and social and occupational functioning at 6-month follow-up; and return-to-work measured as proportion in work at 12-month follow-up.

**Results:**

631 individuals randomized. INT showed higher proportion in work compared with both SAU and MHC at the 12-month follow-up. We found no differences regarding return-to-work time at either the 6- or 12-month follow-up. No differences in symptoms between SAU, MCH or INT were detected, but MHC and INT showed lower scores on Cohen’s perceived stress scale compared with SAU at 12-month follow-up.

**Conclusions:**

Although INT did not hasten return-to-work, it yielded higher proportion in work compared with MHC and SAU.

**Disclosure:**

No significant relationships.

